# Integrative Management of Premenstrual Dysphoric Disorder: The Role of Traditional Chinese Medicine

**DOI:** 10.1155/da/1262387

**Published:** 2026-07-10

**Authors:** Mengbai Xu, Jiaxu Chen

**Affiliations:** ^1^ Department of Traditional Chinese Medicine, The Affiliated Hospital of Qingdao University, Qingdao, Shandong Province, China, qdu.edu.cn; ^2^ School of Traditional Chinese Medicine, Beijing University of Chinese Medicine, Beijing, China, bucm.edu.cn

**Keywords:** conventional medicine, neuroendocrine mechanisms, premenstrual dysphoric disorder, traditional Chinese medicine

## Abstract

Premenstrual dysphoric disorder (PMDD) is a severe form of premenstrual syndrome (PMS), affecting 3%–8% of women of reproductive age. It is characterized by emotional, behavioral, and physical symptoms during the luteal phase, primarily driven by neuroendocrine sensitivity to hormonal fluctuations and dysregulation of central neurotransmitter systems. Genetic predisposition and environmental stressors contribute to its onset. Although conventional treatments such as selective serotonin reuptake inhibitors (SSRIs) and hormonal therapies offer symptom relief, they are often accompanied by adverse effects and limited long‐term efficacy. Traditional Chinese medicine (TCM) posits that the core pathogenesis of PMDD lies in the dysfunction of liver’s conveyance and dispersion, which may consistent with modern biological research evidence. TCM approaches, represented by Chinese herbal medicine and acupuncture, may alleviate symptoms of PMDD by targeting and modulating its core modern pathogenic mechanisms. With its few side effects and the unique advantage of individualized treatment based on syndrome differentiation, TCM holds the potential to become a mainstream therapeutic option for PMDD. This narrative review synthesizes evidence from clinical trials, animal and pharmacological studies to explore the efficacy and underlying mechanisms of TCM in the treatment of PMDD and compares these approaches with conventional therapies. Nonetheless, the integration of TCM into mainstream practice for PMDD is still hampered by challenges such as insufficient evidence‐based medical data. Research in TCM focusing on biomarker identification, establishment of standardized treatment protocols, and refinement of efficacy evaluation systems is expected to drive its evolution into a mainstream treatment option for PMDD.

## 1. Introduction

Premenstrual dysphoric disorder (PMDD) is a severe form of premenstrual syndrome (PMS) that significantly impairs quality of life and, in severe cases, increases the risk of psychiatric comorbidities and suicidal behavior [1]. PMDD, characterized by intense emotional and physical symptoms during the luteal phase of the menstrual cycle, is recognized in the Diagnostic and Statistical Manual of Mental Disorders, Fifth Edition (DSM‐5) as a distinct mood disorder, affecting an estimated 3%–8% of women of reproductive age [2–4].

The exact etiology of PMDD is unclear; genetic predispositions and environmental factors both seem to contribute to its development [5]. From a pathophysiological perspective, the disorder is mainly driven by cyclical hormone fluctuations, which lead to neurobiological disruptions, particularly involving estrogen, progesterone and their interaction with neurotransmitter systems like serotonin (5‐HT) and gamma‐aminobutyric acid (GABA) [6, 7]. The bidirectional crosstalk between reproductive hormones and neurotransmitter pathways underscores the importance of a neurobiological framework in understanding PMDD‐associated depression [4, 6].

Current management strategies for PMDD encompass pharmacological and psychotherapeutic approaches. Selective serotonin reuptake inhibitors (SSRIs) and hormonal therapies like oral contraceptives have shown effectiveness in alleviating PMDD symptoms, but they have limitations, including side effects and contraindications [2, 8, 9]. Such safety issues undermine patient adherence, thus restricting clinical effectiveness. Psychotherapeutic interventions, especially cognitive–behavioral therapy (CBT), provide additional benefits by targeting the cognitive and emotional aspects underlying PMDD [7, 9, 10]. Nevertheless, such a therapeutic strategy does not sufficiently consider the neuroendocrine dysregulation that is naturally present in the disorder. The limitations of existing management strategies have thus compelled an investigation into the use of complementary and integrative therapy options that are safer and more effective.

The use of traditional Chinese medicine (TCM) has been widely applied to the clinical treatment of PMDD in China. With the holistic paradigm of TCM, which focuses on dynamic balance, there is huge potential for it to guide the creation of alternate therapeutic strategies in PMDD. The dysfunction of liver conveyance and dispersion is considered to be one of the main pathogenic factors of PMDD in TCM [11]. Its biological mechanisms are probably related to neuroendocrine dysfunction. Other commonly employed TCM treatments against PMDD, including herbal formulas and acupuncture, could hence have their therapeutic actions in regulating particular neuroendocrine pathways that are involved in PMDD pathogenesis.

Currently, TCM‐based treatment studies related to PMDD generally separate clinical efficacy studies and mechanistic research, leaving minimal full‐scale reviews that combine clinical efficacy information with critical evaluation of the possible neurobiological mechanisms of effects underlying TCM interventions. To bridge this research gap, this narrative review aims to critically evaluate and synthesize the clinical evidence on the effectiveness of TCM interventions in alleviating the core symptoms of PMDD, along with neurobiological mechanisms, in an integrated manner, thereby clarifying the therapeutic mechanisms of TCM interventions in PMDD.

## 2. Methodology

A comprehensive literature search across PubMed and the Web of Science covering publications from 2000 to 2025 was performed to find relevant studies on PMDD. The search covered its clinical profile, etiology and pathophysiology, neuroendocrine mechanisms, and treatments, with a focused retrieval on TCM interventions. This aimed to include both international and Chinese research.

Search terms were developed using a combination of Medical Subject Headings (MeSH) and free‐text terms to cover the core domains of PMDD. For PMDD, terms like “Premenstrual Dysphoric Disorder” and “PMDD” were used. For clinical profile, terms such as “definition,” “diagnostic criteria,” “clinical manifestations,” “prevalence,” “symptomatology,” and “disease burden” were used. For etiology and pathophysiology, terms such as “genetic susceptibility,” “environmental stressors,” and “neurobiological mechanisms” were used. For neuroendocrine mechanisms, search terms such as “hormonal fluctuations,” “neurotransmitter regulation,” “serotonin,” and “neuroinflammation” were used. For treatments, the keywords included “conventional therapy,” “pharmacological interventions,” “selective serotonin reuptake inhibitors,” “hormonal treatment,” and “psychotherapeutic treatment.” For TCM, terms like “Traditional Chinese Medicine”, “herbal medicine”, and “acupuncture” were applied. Boolean operators (AND/OR) were applied to combine these terms for targeted retrieval.

The included studies were original research, reviews, or meta‐analyses centered on PMDD or PMS with documented PMDD‐relevant symptoms. Mechanism‐related studies were included if they provided experimental or observational data on the neuroendocrine basis of PMDD or PMS. Treatment‐focused ones should cover either conventional treatments and their limits or TCM‐based interventions. Studies were excluded if they were meeting abstracts, duplicate publications, or non‐peer‐reviewed literature.

## 3. Clinical Profile

### 3.1. Definition and Diagnostic Criteria

PMDD represents a severe subtype of PMS. It is also classified as a separate psychiatric disorder by the main mental health organizations, such as the American Psychiatric Association (APA) [12]. The disorder has been characterized by significant and cyclically recurring affective and somatic symptoms that appear in the phase of the luteal cycle and disappear soon after menstruation. These symptoms cause significant impairment in social, occupational, or interpersonal functioning and require psychiatric intervention.

According to the DSM‐5 diagnostic criteria, a diagnosis of PMDD requires that an individual experiences at least five symptoms during the luteal phase of the menstrual cycle, with at least one being a core affective disturbance—such as marked mood lability, irritability or anger, depressed mood, or anxiety—as central features [12, 13]. In addition, physical symptoms such as breast tenderness and headache are often present, but emotional symptoms are paramount [12]. These symptoms should be confirmed through prospective daily ratings over at least two symptomatic cycles [6]. A significant component of the diagnostic process now involves ruling out co‐occurring mood disorders that could confound diagnosis, such as major depressive disorder or anxiety disorders, ensuring that the symptoms are specific to the luteal phase [14]. The inclusion of PMDD in the DSM‐5 marks a significant advancement in acknowledging the disorder’s legitimacy, provides clinicians with a standardized framework for diagnosis, and guides evidence‐based therapeutic interventions [13].

### 3.2. Prevalence and Impact

The prevalence of PMS ranges from 20% to 30%, while PMDD affects 3% to 8% of females, highlighting its impact on a portion of the female population [3, 15]. This prevalence is notably consistent across high‐, low‐, and middle‐income countries [16].

Women with PMDD frequently experience significant disruptions in their interpersonal relationships, occupational performance, and daily activities. These disruptions often lead to increased healthcare costs and societal burdens caused by productivity loss and absenteeism [3]. Furthermore, the condition is associated with an elevated risk of comorbid conditions such as depression and anxiety, which can exacerbate the impairment experienced by these women and complicate the diagnosis and treatment [17]. This underscores the need for effective diagnostic and therapeutic interventions to reduce these burdens.

### 3.3. Clinical Manifestations and Symptomatology

#### 3.3.1. Emotional Symptoms

Emotional disturbances are the core feature of PMDD, manifesting as severe irritability, depressed mood, anxiety, and emotional tension that peak premenstrually [2, 18]. These emotional disturbances can be so severe that they significantly impair daily functioning and interpersonal relationships, which differentiates PMDD from the less severe PMS [19]. Emotional symptoms in PMDD vary among individuals; for instance, some women might experience predominant anxiety, while others may exhibit prominent depressive features [20]. Clinically, the symptomatology of PMDD is assessed through a variety of validated instruments, including the daily record of severity of problems (DRSP) [21].

#### 3.3.2. Behavioral Symptoms

In individuals with PMDD, behavioral symptoms constitute a significant component of the clinical presentation and frequently include social withdrawal, decreased motivation, impaired concentration, and a marked reduction in routine activity engagement [22]. These symptoms often occur alongside somatic and affective symptoms, increasing functional impairment and leading to a vicious cycle of emotional distress and behavioral avoidance. Effective clinical management of behavioral symptoms requires a multidimensional and individualized strategy that emphasizes both symptom control and the enhancement of patient agency to optimize long‐term outcomes and prevent chronic impairment [23, 24].

#### 3.3.3. Physical Symptoms

Physical symptoms in PMDD represent a significant clinical and functional burden, commonly presenting as breast tenderness, headaches, bloating, and muscle or joint pain [18]. These manifestations exacerbate the overall distress experienced by patients and are critical to understanding the full impact of PMDD on physical health and quality of life [25, 26]. Given the substantial overlap with conditions such as primary dysmenorrhea and migraine, a thorough clinical evaluation requires an integrated approach encompassing detailed history‐taking, prospective symptom monitoring, and systematic differential diagnosis [27–29]. Although lifestyle modifications, such as increased physical activity and dietary changes, can alleviate some of the physical discomforts associated with PMDD, pharmacotherapy remains a cornerstone for patients with severe symptoms [30].

## 4. Etiology and Pathophysiology

### 4.1. Genetic and Environmental Influences

PMDD has a multifactorial etiology, in which genetic susceptibility plays a critical role. Evidence from family and twin studies suggests moderate‐to‐high heritability [31]. Specific genetic variants predispose individuals to mood disorders closely related to PMDD. For instance, polymorphisms in genes such as 5HTTLPR, which influences serotonin transport, have been implicated in affective disorders, indicating a possible genetic susceptibility pathway [32]. Additionally, GABRB2 copy‐number variations have been associated with PMDD in multiethnic cohorts, pointing to GABAergic involvement [33].

Environmental influences, particularly during formative years, play an equally pivotal role. Early life stressors such as childhood adversity, abuse, and familial dysfunction significantly predispose individuals to PMDD by affecting hypothalamic–pituitary–adrenal (HPA) axis function and allopregnanolone (ALLO) metabolism during the luteal phase [34].

The influence of exposure to adverse psychosocial environments interacts with genetic predispositions in a complex manner. Gene–environment interactions (GxE) are pivotal in determining the phenotypic expression of PMDD, as they mediate the genetic susceptibility to environmental stressors [35, 36]. Studies have found that the effect of brain‐derived neurotrophic factor (BDNF) Val66Met varies depending on levels of hormone exposure, suggesting a GxE mechanism modulating stress reactivity [37]. Moreover, epigenetic mechanisms such as DNA methylation and histone modification resulting from such interactions indicate a biological basis for stress sensitization, making PMDD symptomatic in stress‐prone environments [38, 39].

**Figure 1:**
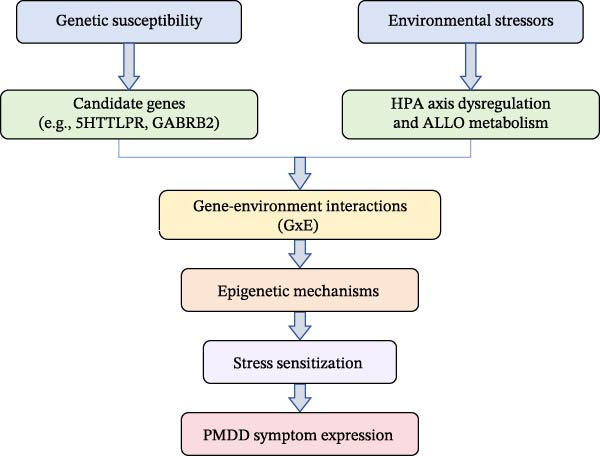
Gene–environment interaction in PMDD etiology.

The interplay between genetic predispositions and environmental factors in the etiology of PMDD is illustrated in Figure 1.

### 4.2. The Neurobiological Basis of PMDD

#### 4.2.1. Hormonal Sensitivity and Neuroendocrine Dysregulation

Fluctuations in gonadal hormones, particularly estrogen and progesterone, play a pivotal role in the onset and persistence of PMDD by closely linking to mood changes in susceptible individuals [40]. These hormones are not only pivotal in regular menstrual regulation but also influence brain function, impacting regions tied to mood regulation such as the limbic system and prefrontal cortex [41]. Estrogen tends to exert a protective effect against depression by modulating neurotransmitters such as 5‐HT, dopamine (DA), and GABA, thereby enhancing mood. However, declining progesterone levels, common in the luteal phase, can destabilize the mood, heightening the risk of depression. Neuroactive metabolites of progesterone, particularly ALLO, act as positive allosteric modulators of GABA_A receptors and have been implicated in affective lability and mood dysregulation during the luteal phase, especially in hormonally sensitive individuals [42].

The interaction between hormonal changes and mood is further elucidated by observing the patterns of mood changes across different reproductive life stages, such as postpartum and perimenopausal periods [43]. This pattern suggests that hormonal fluctuations can trigger mood disorders and reflect a common neurobiological framework in various reproductive‐related depressions. The efficacy of hormonal therapies provides clinical corroboration for this. However, the precise mechanisms involved remain complex and are modulated by genetic susceptibility and environmental factors [44]. Therefore, ongoing research is necessary to elucidate the exact pathways and interactions impacted by hormonal changes, aiding in the development of targeted therapies.

#### 4.2.2. Serotonin and Other Neurotransmitter Involvement

Serotonin plays a pivotal role in the modulation of mood and emotional stability within the spectrum of neurotransmitter disturbances observed in PMDD [4]. Women with PMDD show altered serotonergic functioning during the luteal phase, which correlates with worsening depressive symptoms [45, 46]. Abnormalities in tryptophan metabolism, serotonin transporter function, and reduced receptor sensitivity all affect these critical mood‐regulation pathways [45–47]. The significant efficacy of drugs targeting this pathway, such as SSRIs, has further confirmed the key role of the serotonin system in the pathogenesis of PMDD [23, 48].

Other neurotransmitters, including GABA and glutamate, contribute to PMDD‐associated depression neurobiology [49]. Dysregulation of the GABAergic system, linked to ALLO, impairs inhibitory control over HPA axis activation, contributing to heightened stress sensitivity and emotional instability [43, 50]. Additionally, studies have found that patients with PMDD exhibit disrupted glutamate–glutamine homeostasis in limbic brain regions during the luteal phase, suggesting that such glutamatergic dysregulation may synergize with impaired GABAergic inhibition to further exacerbate mood disturbances [49]. Notably, imbalances in these neurotransmitter systems are intricately intertwined with genetic and environmental factors [51]. Therefore, future research should aim to elucidate these multidimensional interactions in greater depth to inform the development of more refined and individualized interventions for PMDD.

**Figure 2:**
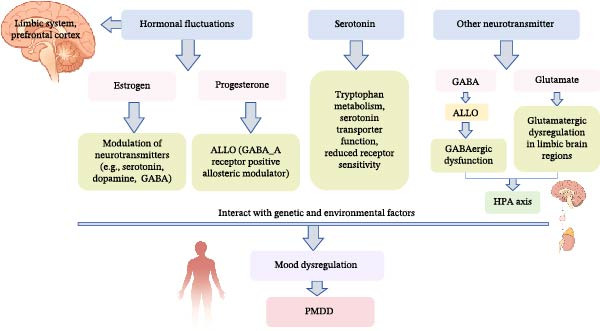
The neurobiological basis of PMDD.

The neurobiological basis of PMDD is summarized in Figure 2.

## 5. Conventional Therapies

### 5.1. Pharmacological Interventions

Current pharmacological approaches to PMDD primarily target neurotransmitter function and hormonal regulation due to their key roles in the disorder’s pathophysiology. SSRIs are often the first line of therapy used to treat PMDD due to their role in ameliorating mood symptoms efficiently [9, 52, 53]. Clinical trials have consistently demonstrated the efficacy of SSRIs such as fluoxetine, sertraline, and paroxetine in the treatment of both emotional and physical symptoms associated with PMDD [6, 23]. These medications exert their therapeutic effects by increasing serotonin availability in the synaptic cleft [53]. For SSRIs, the luteal phase dosing strategy ensures efficacy while minimizing the burden associated with long‐term continuous medication use [54]. Furthermore, serotonin–norepinephrine reuptake inhibitors (SNRIs), such as venlafaxine, offer a viable alternative for patients who demonstrate an inadequate response to SSRIs [52, 55].

Apart from antidepressants, hormonal interventions form another pillar in the pharmacological management of PMDD [3]. Combined oral contraceptives (COCs), particularly those containing drospirenone, alleviate PMDD symptoms by stabilizing hormonal fluctuations [9, 23]. In more refractory cases, gonadotropin‐releasing hormone (GnRH) agonists may be employed to induce a hypoestrogenic state [23]. However, this approach necessitates caution due to the risk of reduced bone mineral density and menopausal symptoms, thereby limiting its use to short‐term application or as an adjunctive strategy combined with add‐back therapy [56]. Given the significant interindividual variability in treatment response, the importance of personalized medicine is underscored, warranting a tailored pharmacotherapeutic approach in PMDD that optimally balances efficacy and tolerability.

### 5.2. Psychotherapeutic Strategies

While pharmacological treatments are central to managing PMDD, psychotherapeutic interventions provide complementary strategies to address the psychological stress and behavioral symptoms associated with the disorder. CBT offers a structured, time‐limited psychological intervention [57]. Clinicians frequently use CBT to help patients identify and change maladaptive thought patterns and behaviors that worsen PMDD symptoms [58]. Through CBT, patients are equipped with coping mechanisms and cognitive restructuring techniques, which are pivotal in reducing anxiety and depression symptoms during the luteal phase [59].

### 5.3. Efficacy and Limitations of Current Treatments

While SSRIs are the first‐line pharmacological treatment for PMDD, they may induce adverse effects, including nausea, headache, somnolence, and sexual dysfunction [60]. These side effects may compromise long‐term adherence. Additionally, a subset of patients exhibits a suboptimal initial response, necessitating adjunctive therapies or dose adjustments. In hormonal therapy, COCs alleviate symptoms by suppressing ovulation and stabilizing estrogen levels, but they may cause breakthrough bleeding or withdrawal symptom fluctuations, which can disrupt treatment continuity [61, 62]. Safety considerations, such as the risk of thrombosis, necessitate caution when prescribing COCs to susceptible individuals [63]. GnRH agonists, which induce chemical ovarian suppression, are effective for severe PMDD but are limited by significant drawbacks. Long‐term use is associated with bone mineral density loss and menopausal‐like symptoms [56].

CBT is a commonly used psychological intervention for PMDD. When used as an adjunct to SSRIs, CBT’s efficacy is comparable to that of pharmacotherapy alone [9]. Nevertheless, CBT has some notable limitations. As a standalone treatment, it is less effective for severe PMDD, often requiring pharmacological support to stabilize acute symptoms [64]. The gradual nature of CBT’s therapeutic effects may delay symptom relief, posing challenges to patient adherence [9].

Current treatments for PMDD demonstrate substantial efficacy but are constrained by significant limitations. Pharmacological interventions, while effective, are hindered by adverse effects and risks that necessitate careful patient selection and monitoring. Psychological interventions like CBT offer valuable adjunctive benefits but are less effective as monotherapies for severe cases. The integration of pharmacological and psychological approaches holds promise, yet optimal combination strategies remain underexplored. Future research should focus on exploring treatments for PMDD that are effective with minimal adverse effects, improving adjunctive treatment options, and addressing access and adherence barriers to psychological interventions to enhance comprehensive PMDD management.

## 6. TCM Approaches

### 6.1. Theoretical Foundations

#### 6.1.1. Core Theories

TCM boasts a venerable heritage in managing mood disorders through a holistic framework that encompasses physical, emotional, and spiritual dimensions. As early as several 1000 years ago, classical Chinese medical texts such as the *Huangdi Neijing* explicitly recognized the pivotal role of emotional disturbances in the onset and progression of disease. The TCM perspective on emotional and mental wellness revolves around the concept of an integrated framework of the Five Elements and the Zang‐Fu theories [65].

From a TCM perspective, mental health is viewed as the reflection of the body’s internal harmonies. The core of this theory is the harmonious flow of Qi (vital energy), the dynamic balance of Yin‐Yang, and the coordinated performance of the internal Zang‐Fu. According to practitioners, the emotional state of people has a close connection to the physiological state of some organs, such as the liver (a functional system in TCM, not the anatomical liver), related to anger. It is a holistic perspective that focuses on the concept of the inseparability of mind and body, stating that emotions cannot be considered as epiphenomenons that are separated from the physical body but as external signs of the functional status of the Zang‐Fu, Qi, and blood.

#### 6.1.2. Etiology and Pathogenesis

In TCM, emotions are perceived to play a dual purpose, which is both as a causative agent and as a clinical sign of disease. Specifically, emotional disturbances disrupt the flow of Qi and the balance of Yin‐Yang, leading to disharmonies in Zang‐Fu and manifesting as clinical anxiety, depression, insomnia, or cognitive decline. The liver’s role in maintaining the smooth flow of Qi is particularly crucial, as stagnant or invasive Liver Qi is believed to lead to irritability, depression, and mood disorders [66–68]. An epidemiological study indicates that the pathogenesis of PMDD mainly involves the dysfunction of liver’s conveyance and dispersion [11]. In the context of PMDD, excessive dispersion and spreading of Liver Qi can trigger Liver Qi invasion, manifesting as severe irritability and physical symptoms such as headaches. Nevertheless, insufficient conveyance and dispersion of Liver Qi may cause Liver Qi stagnation, which is indicated by depression and behavioral symptoms like social withdrawal. Both Liver Qi invasion and Liver Qi stagnation have been commonly observed clinical syndromes of PMDD in TCM [11]. In the Five Elements theory view, restrained Liver Qi is prone to invade the spleen, causing the syndrome of liver stagnation and spleen deficiency, producing physical symptoms of breast tenderness and bloating and behavioral symptoms such as a marked reduction in routine activity engagement. Moreover, since the liver has the role of storing blood and regulating menstruation in TCM, cyclic changes in Qi and blood throughout the luteal phase may make women more vulnerable to Liver Qi stagnation and thus bring on the symptoms of PMDD.

#### 6.1.3. Potential Modern Biological Correlates of TCM Pathogenesis

In accordance with TCM theory, mental disorders are often caused by imbalances or blockages of the liver Qi. The conceptualization is different as compared to the biomedical model that commonly attributes mental disorders to neurochemical imbalances in the brain. Modern scientific studies provide preliminary evidence supporting the potential biological correlates of TCM pathogenesis in PMDD. Evidence from animal experiments suggests a potential link between the biological basis of the Liver Qi Invasion subtype of PMDD and ALLO‐mediated dysregulation of GABAA‐Rα4 expression in emotion‐related brain regions, including the amygdala and hippocampus [66]. Since Liver Qi stagnation is a similar phenomenon between PMS and PMDD, studies on PMS offer much‐needed information about the biological basis of PMDD. In particular, as it has been shown, the augmentation of the µ‐opioid receptor (MOR) expression and cAMP/ERK signaling pathway activation in the hippocampus is greatly associated with the development of this syndrome [69]. Moreover, the signs of depression in the context of Liver Qi stagnation might also be linked to reduced levels of pregnenolone and ALLO, increased amounts of dehydroepiandrosterone (DHEA), and higher ratios of DHEA/ALLO and DHEA/pregnenolone throughout the luteal phase [70].

Despite the scarcity of biological research that examines the concept of TCM pathogenesis in PMDD, these findings demonstrate that there is a crosstalk between the doctrine of “Liver governing conveyance and dispersion” and the central nervous system, which justifies the potential efficacy of measures designed to soothe Liver Qi and balance Yin‐Yang. Specifically, their observations support that TCM treatments presumably reduce PMDD symptoms through the restoration of neurotransmitter receptor signaling and thus eliminate the underlying pathological disruptions.

### 6.2. Therapeutic Approaches

#### 6.2.1. Chinese Herbal Medicine

TCM offers a rich repertoire of herbal treatments specifically designed to address the symptoms associated with PMDD. According to a study, the most frequently used TCM herbal formulas in PMS/PMDD are Xiaoyao San and Chaihu Shugan San [71]. These classic formulas are commonly used to regulate Liver Qi in TCM clinical practice. Among these, Xiaoyao San, a classic formula for the syndrome of liver depression and spleen deficiency, is composed of Bupleurum root (Chai Hu), *Angelica sinensis* (Dang Gui), White Peony root (Bai Shao), and other herbs, which serve to regulate the Liver Qi, strengthen the Spleen Qi, and nourish the blood. Pharmacological studies have shown that the mood‐regulating effects of Xiaoyao San may involve multiple mechanisms, including modulation of monoamine neurotransmitters (e.g., 5‐HT and DA), regulation of the HPA axis, enhancement of synaptic plasticity, upregulation of neurotrophic factors, biological regulation in the arcuate nucleus of the hypothalamus, and regulation of hippocampal neuroplasticity [72–76]. Chaihu Shugan San is frequently recommended for its dual capacity to regulate liver dysfunction and relieve emotional agitation, which aligns with the TCM theoretical foundations that emotional disturbance is often rooted in the liver’s dysfunction [71]. Chaihu Shugan San modulates mood disorders through a multifaceted mechanism. Network pharmacology and preclinical studies indicate that Chaihu Shugan San regulates monoaminergic neurotransmitters, modulating the HPA axis, enhancing synaptic plasticity, upregulating neurotrophic factors such as BDNF, and promoting hippocampal neurogenesis and angiogenesis [77–81]. Regardless of these mechanistic observations, well‐conducted randomized controlled trials (RCTs) focusing on these classic formulas alone in PMDD are scarce. The available clinical evidence is mostly made up of observational studies, which tend to have small sample sizes and methodological differences. Given that PMDD and PMS share a consistent TCM etiology and pathogenesis, findings from TCM studies targeting PMS can be reasonably extrapolated to inform the treatment of PMDD. Jiawei Xiaoyao Pill is a modified version of Xiaoyao San with additional herbs to clear blood heat. A multi‐center RCT involving 144 patients demonstrated that after three menstrual cycles of treatment, Jiawei Xiaoyao Pill significantly reduced luteal‐phase DRSP scores compared to placebo in PMS (liver depression, spleen deficiency, and blood‐heat syndrome) patients, exhibiting favorable safety and tolerability [82].

Additionally, proprietary herbal formulations that regulate Liver Qi, such as Jingqianping and Jingqianshu granules, have shown efficacy in treating PMDD‐like emotional symptoms in animal models. In luteal‐phase‐isolated macaques, Jingqianping treatment ameliorated PMDD‐like behavioral disturbances, restored serum levels of 5‐HT, DA, and norepinephrine, and normalized estradiol and progesterone concentrations [83]. Jingqianping Granule is used to treat the shared TCM syndrome of Liver Qi invasion in both PMS and PMDD. A mechanistic study in rat models indicates that its therapeutic effects involve the region‐specific modulation of MORs, characterized by upregulation in the cerebral cortex of parietal and frontal lobes and downregulation in the hypothalamus and hippocampus [84]. Jingqianshu granules can alleviate premenstrual depressive symptoms in the model group of macaques and significantly reduce the elevated levels of serum prolactin and monoamine neurotransmitters [85]. The preclinical experiments show that Jingqianshu granules have antidepressant‐like effects via multi‐target actions which include the orexin system in the hypothalamus‐amygdala, inflammatory cytokines, and complex interactions within the basolateral amygdala (BLA), including increasing the concentration of the neurosteroid ALLO and serotonin, as well as normalization of the neuronal electrophysiological activity [86, 87]. The clinical usefulness of Jingqianshu granules has been shown in a recent RCT that included 156 women with PMS liver qi stagnation type [88]. Following three treatment cycles, Jingqianshu granules demonstrated a clinically meaningful reduction in the DRSP score compared to that of placebo (Cohen’s *d* = −0.51). The improvement of TCM syndrome scores and core individual symptoms such as anxiety and emotional instability was also statistically significant, and no additional risk to adverse events was observed. Despite the fact that the trial was conducted under the category of PMS, the common pathophysiological processes and TCM pathogenesis between both ailments justify using these results as valuable clinical evidence concerning the effectiveness of Jingqianshu pills in treating PMDD.

#### 6.2.2. Acupuncture

Acupuncture, a widely utilized modality within TCM, has garnered increasing attention for its potential efficacy in alleviating PMDD symptoms. It is practiced by penetrating the minute needles into particular acupoints in order to relieve sensations by the adjustment of Qi, blood, and meridians. Preclinical evidence shows that the employment of acupuncture in the form of electroacupuncture counteracts emotional behaviors through the effect of the regulation of monoamine neurotransmitters and the HPA axis, the down‐regulation of amygdala CRH/CRHR1 signaling, the neuroplasticity and neuroprotection, the reduction of the inflammatory response, and the sense of neuroendocrine regulation [89, 90]. The complex effects are probably what lead to the reduction in emotional symptoms of PMDD. One of the key acupoints used in regulating Liver Qi is Taichong (LR3). Recent studies have proven that stimulating this acupoint relieves depressive‐like behavior by increasing the synaptic activity within the hippocampal vCA1 region, and the mechanisms behind it include increased AMPA receptor trafficking and BDNF expression [91].

Clinically, acupuncture may reduce the overall clinical severity in PMS patients and improve emotional and physical symptoms associated with PMDD at the end of the intervention [92]. The findings are based on a single‐blind RCT conducted on 30 women who have PMDD and have shown that true acupuncture treatment performed in two menstrual cycles significantly lowered scores of the Hamilton Anxiety (HAMA) and Hamilton Depression (HAMD) Rating Scales compared to sham acupuncture [93]. A meta‐analysis demonstrated that acupuncture is effective in alleviating PMDD symptoms irrespective of the timing of intervention initiation, with specific acupoints—particularly Sanyinjiao (SP6), Taichong (LR3), and Guanyuan (RN4)—identified as the most frequently used and potentially efficacious targets in clinical protocols [94]. A systematic review of 19 RCTs, including eight acupuncture studies, concluded that acupuncture and moxibustion interventions resulted in a ≥50% reduction in PMS/PMDD symptom severity, with no serious adverse events reported [95]. These findings suggest that acupuncture may serve as an effective, minimally invasive, well‐tolerated, and safe treatment option for both emotional and physical premenstrual symptoms.

#### 6.2.3. Other Therapeutic Interventions

Beyond herbal medicine and acupuncture, TCM offers a diversified intervention spectrum, including TCM manual therapies, mind–body interventions, and lifestyle adjustments. Manual TCM therapies such as acupressure have been found to be clinically efficacious and safe when it comes to improving PMS/PMDD symptoms in comparison to no treatment or routine care [92]. Mind‐body interventions such as Tai Chi and Qigong, which aim to harmonize Qi flow and rebalance Yin‑Yang, have demonstrated beneficial effects on mood and stress regulation in chronic conditions [96]. The literature indicates that Tai Chi and Qigong regulate the autonomic nervous system and inhibit stress connected with HPA axis reactivity. Their regulatory mechanisms on mood are likely to include regulating various areas of the prefrontal cortex, the limbic system, and the striatum or changing the gene expression of inflammatory signals and stress‐sensitive pathways [97]. Though PMDD‐specific trials are sparse, these practices may offer adjunctive benefits in emotional regulation during the premenstrual phase. In addition, lifestyle modifications are an important component of TCM‐based interventions for PMDD, aligning with clinical practice guidelines for PMDD management issued by professional organizations such as the American College of Obstetricians and Gynecologists (ACOG) [98]. Based on the holistic concept of TCM, the aim of all the above mentioned interventions is to restore internal harmony and achieve physical–mental integration.

Table 1 summarizes common TCM treatments for PMDD.

**Table 1:** Summary of traditional Chinese medicine interventions for PMDD.

TCM approach	Chinese herbal medicine	Acupuncture	Other therapeutic interventions
Intervention method	Classic formulas: Xiaoyao San and Chaihu Shugan San Proprietary formulations: Jingqianping and Jingqianshu granules	Specific acupoints: SP6, LR3, and RN4 Electroacupuncture Moxibustion	Manual therapies: acupressure Mind‐body interventions: Tai Chi and Qigong Lifestyle adjustments
TCM core principle	Regulating Liver Qi	Harmonizing Qi and blood Dredging Meridians	Restoring internal harmony Achieving physical–mental integration
Mechanism	Regulating monoamine neurotransmitters, HPA axis, neurosteroids, GABA system, hypothalamic‐amygdala orexin system, inflammatory factors, MOR, and hormone levels Enhancement of synaptic plasticity and neurotrophic factors	Regulating central/peripheral nervous system, neuroendocrine systems, and monoamine neurotransmitters Inhibiting amygdala CRH/CRHR1 signaling Enhancing synaptic function, Reducing inflammation	Modulating multiple prefrontal regions, the limbic system, and the striatum Affecting inflammation/stress‐related gene expression
Clinical research	Jiawei Xiaoyao Pill (modified Xiaoyao San, multi‐center RCT, *n* = 144, PMS); Jingqianshu granule (RCT, *n* = 156, PMS) significantly reduce DRSP scores (Daily Record of Severity of Problems), with efficacy and safety extrapolable to PMDD	A single‐blind RCT (*n* = 30, PMDD population) shows that it can reduce HAMA and HAMD scores Meta‐analyses and systematic reviews confirm that acupuncture/moxibustion relieves symptoms by ≥50% without serious adverse events	Systematic review with meta‐analysis (*n* = 351, PMS/PMDD) shows acupressure can reduce PMS scores compared with no treatment/routine care without serious adverse events

## 7. Methodological Quality Assessment

The included studies differ in terms of methodological quality. The overall design quality of conventional medicine studies is relatively high. More RCTs employ strict randomization, blinding, and standardized outcome indicators. However, most clinical trials include a limited sample size. Many of them are single‐center studies, which may introduce potential bias and limit their universal applicability. In contrast, studies on TCM are usually less methodologically rigorous. Most studies have a small sample size and a single‐center design, lacking strict blinding or allocation concealment. There are significant differences between the TCM syndrome diagnostic standards and herbal formulations. There are few RCTs of herbal formulations for PMDD, and much evidence is inferred from the studies of PMS. Animal experiments mainly focus on liver qi stagnation models, with limited translational value to human pathophysiology. The evidence quality of TCM studies on PMDD treatment varies widely from low to high, limiting the strength of clinical recommendations.

Although conventional medicine studies provide more rigorous design control and mechanistic evidence, both conventional and TCM‐related mechanistic studies of PMDD rely heavily on animal models and human studies with limited sample sizes. Most of the evidence obtained is of low to medium certainty. In conventional medicine and TCM studies, diagnostic criteria and evaluation tools are not completely unified. This hinders cross‐study comparison and highlights the need to improve methodological standardization.

## 8. Discussion

### 8.1. Current Status of TCM Treatment

In the context of the holistic framework of TCM, PMDD is seen as an expression of the imbalance of homeostasis between Qi, Yin‐Yang, and Zang‐Fu. The core pathology is the dysfunction of the liver’s conveyance and dispersion, followed by the blockage of Liver Qi flow and imbalance of Qi and blood. The discovery of the mechanisms of GABA receptors, MOR receptors, and dysfunction of neurosteroid hormones has given it a biological explanation of the TCM pathogenesis of PMDD, adding value to the scientific foundation of the TCM theory.

Clinically, TCM approaches to cure PMDD are diverse. The herbal formulas and acupuncture are the main treatment modalities which are frequently supplemented by manual therapy, mind–body interventions, and lifestyle changes. The essence of therapy is to regulate Liver Qi, balance Qi and blood, and restore internal harmony. Mechanistic investigations of modern studies demonstrate that the mechanism of action underlying TCM in the treatment of PMDD is through the regulation of neurotransmitters and neuroendocrine systems and improvement of synaptic plasticity, among others. Clinical trials, systematic reviews, and meta‐analyses have provided evidence that TCM interventions are useful and safe to alleviate the symptoms of PMDD and improve the quality of life of the patients substantially.

### 8.2. Comparative Analysis of TCM and Conventional Therapies

TCM adopts a holistic, individualized approach to PMDD management, contrasting with the symptom‐focused strategies of conventional medicine. Conventional medicine typically relies on pharmacological interventions targeting specific neurotransmitter or hormonal changes believed to underlie PMDD. TCM conceptualizes PMDD as a manifestation of internal imbalances, particularly involving Liver Qi stagnation and disharmony of Qi and blood. Treatment in TCM typically incorporates herbal formulas and acupuncture to restore homeostasis and promote emotional stability [99].

Each approach offers unique therapeutic strengths, but they differ significantly in terms of clinical benefits and safety profiles. Current mainstream treatments, including SSRIs and hormonal therapies, have established robust evidence for efficacy and swift symptom relief but often are associated with side effects that may limit treatment adherence [60, 100]. In contrast, TCM approaches, such as the use of specific herbal formulations and acupuncture, tend to have fewer side effects and focus on long‐term health improvement by addressing root causes rather than merely alleviating symptoms [95]. However, TCM interventions typically require longer treatment durations to achieve clinically significant outcomes [71, 101].

Table 2 presents a comparison of common treatments for PMDD between TCM and conventional therapies.

**Table 2:** Comparison of TCM and conventional therapies for PMDD.

Dimension	TCM approach	Conventional therapies
Treatment focus	Holistic, homeostasis	Symptom‐specific, biochemical pathways
Common interventions	Herbal formulas, acupuncture	SSRIs, hormonal therapy
Duration	Variable; often longer‐term	Short‐term to medium‐term
Side effects	Minimal; individualized	Moderate to severe; may limit adherence
Patient preference	Individualized, culturally integrated	Standardized, protocol‐driven

### 8.3. Challenges of TCM Research

The study of TCM is full of challenges, with the major problem being the lack of proper methodologies and inability of the available evidence to conform to the existing mainstream scientific criteria. The majority of the clinical trials of TCM in treating PMDD suffer in terms of their small sample size, absence of control groups, and poor blinding, which reduces the validity and reproducibility of these results, limiting further adoption of TCM in the paradigm of evidence‐based medicine (EBM). Numerous investigators have demanded additional RCTs and longitudinal studies that will follow rigid research methods and will confirm effectiveness of TCM treatments in PMDD. Nonetheless, such change is obstructed by various practical challenges, such as financial limitations and difficulties in modernizing diagnostics of TCM, which could be difficult to match with biomedical diagnostics.

The use of anecdotes and historical documents in TCM also interferes with the development of a sound scientific literature that can be assessed using the perspective of EBM. Although an increasing amount of literature dedicated to TCM has accumulated, most TCM studies on PMDD remain based on classical clinical observations or small case studies that might fail to reach the highest requirements of methodologies considered as high‐quality scientific evidence. In addition, PMDD treatment in TCM is usually individualized, and each specific situation has its particular treatment. Such a variety of PMDD treatments in TCM makes it difficult to create standardized and generally applicable protocols for clinical trials.

Moreover, the absence of standardized procedures to document and quantify the results of TCM in PMDD still represents a significant obstacle to the advancement of TCM research in PMDD. It is vital to create TCM‐specific validated tools and outcome measures to measure the health outcomes correctly in PMDD and provide the quantified and comparable data to the related research. Owing to some key issues in the field of TCM studies mentioned above, its modernization and evidence‐based development have been hindered, and this has become a significant barrier to integrating TCM into the conventional medicine system.

Table 3 lists current challenges and potential improvements.

**Table 3:** Challenges and potential improvements of TCM for PMDD.

Challenge	Current state	Potential improvement
Methodological issues	Small sample sizes, lack of control groups, and insufficient blinding	Adoption of stringent RCT and longitudinal study protocols
Standardization	Insufficient EBM compatibility of research evidence base and personalized treatment variability	Development of standardized and broadly applicable clinical trial protocols
Validation tools	Lack of TCM‐specific outcome measures and quantification systems	Development of TCM‐tailored assessment tools and outcome measures

### 8.4. Barriers to Integrating TCM Into Conventional Medicine

TCM has a long history, with applications spanning over thousands of years. Nevertheless, the introduction of TCM into conventional EBM has remained a complicated and multifaceted barrier, particularly the application of TCM in the treatment of PMDD. The predicament can be partially explained by some of the previously mentioned core issues in TCM research, but it has predominantly to do with the basic disparity in the philosophical background, diagnostic approach, and treatment modalities between TCM and conventional medicine. One of the major barriers is the lack of standardization and consensus within TCM itself, which can result in heterogeneity in diagnosis and treatment approaches, leading to resistance from practitioners who rely on standardized protocols and evidence‐based practices [102]. This issue is equally applicable to TCM in the treatment of PMDD.

Another significant barrier is cultural and educational. Current mainstream medical education traditionally does not include TCM in many countries, which limits understanding and appreciation of its principles among healthcare providers, making it difficult for them to grasp the precise determination of individualized TCM regimens for PMDD. Consequently, there is a shortage of healthcare professionals proficient in both practices, making interdisciplinary communication and collaboration difficult. This cultural gap extends to regulatory frameworks as well, where TCM often lacks the rigorous clinical testing and approval processes that are standard for current pharmaceuticals. As a result, TCM faces challenges in meeting the stringent safety and efficacy criteria required by regulatory bodies such as the U.S. Food and Drug Administration (FDA) and the European Medicines Agency (EMA) [103]. The aforementioned barriers further delay the development and clinical application of integrated TCM and conventional medical treatments for PMDD.

Economic factors also contribute to the slow integration of TCM into conventional practices. The financial incentives associated with pharmaceutical drugs for PMDD often overshadow the adoption of TCM, which is generally considered less financially rewarding due to its holistic and preventive nature. Furthermore, the insurance coverage for TCM treatments is limited in many countries, reducing patient access to these therapies and discouraging their use [103]. This situation presents a barrier to the dissemination of TCM‐specific therapeutic approaches for PMDD.

### 8.5. Opportunities for Future Research and Development

The future of TCM research for PMDD needs to focus on expanding our understanding of the underlying mechanisms and improving the efficacy of treatment modalities. Current treatments often involve a trial‐and‐error approach, which can be time‐consuming and burdensome for patients. Future research should aim to identify biomarkers that predict treatment response, providing a more personalized approach to PMDD management [104, 105]. To achieve progress in this area, collaborative efforts between academia, industry, and clinical practitioners are needed. Such collaborations can facilitate the design of adaptive clinical trials that are more responsive to patient needs and allow for the exploration of various therapeutic strategies concurrently. This adaptive trial design can better reflect real‐world clinical settings and provide more rapid insights into the effectiveness of emerging therapies [105, 106].

To align TCM research on PMDD with EBM standards, innovative methodologies such as N‐of‐1 trials, pragmatic RCTs (pRCTs), and real‐world studies are recommended [100, 107]. It is worth noting that TCM provides a certain direction for the treatment of PMDD. While there are numerous challenges in integrating TCM into modern medical practice, there are also promising opportunities for future research and development. Collaborative efforts between TCM practitioners and conventional medicine researchers could facilitate a more synergistic integration by combining the strengths of both systems.

Future research should explore the research value and translational opportunities of TCM in the field of PMDD. One key opportunity lies in identifying TCM as a source of novel therapeutic agents. Many modern drugs have been derived from plants traditionally used in Chinese medicine, and advancing TCM research requires further elucidation of the polypharmacological mechanisms of herbal medicines, which attests to the vast potential for discovering new pharmaceuticals that could be developed and validated through modern scientific techniques. Additionally, the development of hybrid models incorporating genetic and molecular biology tools could provide deeper insights into the mechanisms underlying TCM. Such efforts could bridge the gap between traditional practices and modern biomedical research, enhancing the scientific understanding of TCM’s clinical benefits.

## 9. Conclusion

Currently, the pathogenesis of PMDD involves multiple neuroendocrine mechanisms. A key limitation of conventional therapies for PMDD is their failure to meet the clinical demands for treatment safety and individualization, which often results in poor patient adherence and suboptimal therapeutic efficacy. Based on the traditional core pathogenesis that lies in the dysfunction of the liver’s conveyance and dispersion, TCM pathogenesis may correspond with the modern biological research evidence of PMDD. TCM approaches represented by Chinese herbal medicine and acupuncture, via targeted modulation of the core pathogenesis of PMDD, exhibit favorable safety and clinical effectiveness in alleviating their clinical symptoms and improving patients’ quality of life. Compared with conventional therapies, TCM has advantages including individualized syndrome differentiation and treatment, along with minimal adverse effects. These attributes fully demonstrate its potential as a complementary clinical approach for PMDD.

Nevertheless, there is still a lack of EBM data due to the poor design of methodologies and high levels of heterogeneity within individualized treatment protocols in studies on the topic of TCM interventions in PMDD. Meanwhile, several factors such as cultural cognitive differences,professional education system gaps, and other factors have prevented the incorporation of TCM into the mainstream diagnosis and treatment model of PMDD. Ongoing TCM research systematically targets major challenges in PMDD care. Key focus areas include biomarker identification, treatment standardization, outcome measure validation, and the integration of TCM with conventional medicine. This sustained research investment will be conducive to advancing TCM from a complementary clinical approach to a valuable mainstream therapeutic option for PMDD.

## Author Contributions

Mengbai Xu conducted the literature search and was responsible for drafting and revising the manuscript. Jiaxu Chen designed the framework of the manuscript and gave final approval of the version to be submitted.

## Funding

This work was supported by the National Natural Science Foundation of China (No. 82405459, No. 82430126) and Qilu Biancang Traditional Chinese Medicine Talent Cultivation Project of Shandong Health Commission (No. [2023]78).

## Disclosure

All authors read and approved the final manuscript.

## Ethics Statement

This review did not require ethical approval as it involved the synthesis of existing studies.

## Conflicts of Interest

The authors declare no conflicts of interest.

## Data Availability

Data sharing is not applicable to this article as no datasets were generated or analyzed during the current study.
